# Perception of Suicide Risk in Mental Health Professionals

**DOI:** 10.1371/journal.pone.0149791

**Published:** 2016-02-24

**Authors:** Tim M. Gale, Christopher J. Hawley, John Butler, Adrian Morton, Ankush Singhal

**Affiliations:** 1 Department of Research, Hertfordshire Partnership University NHS Foundation Trust, Hatfield, United Kingdom; 2 Department of Psychology, University of Hertfordshire, Hatfield, United Kingdom; 3 Department of Post-graduate Medicine, University of Hertfordshire, Hatfield, United Kingdom; 4 School of Health, University of Central Lancaster, Preston, United Kingdom; 5 Reigate Psychology Service, Reigate, Surrey, United Kingdom; 6 Psychological Medicine Service, The Royal Oldham Hospital, Oldham, United Kingdom; University of St Andrews, UNITED KINGDOM

## Abstract

This study employed an independent-groups design (4 conditions) to investigate possible biases in the suicide risk perception of mental health professionals. Four hundred participants comprising doctors, nurses and social workers viewed a vignette describing a fictitious patient with a long-term mental illness. The case was presented as being drawn from a sample of twenty similar clinical case reports, of which 10 were associated with an outcome of suicide. The participant tasks were (i) to decide whether the presented vignette was one of those cases or not, and (ii) to provide an assessment of confidence in that decision. The 4 conditions were used to investigate whether the presence of an associated face, and the nature of the emotional state expressed by that face, affected the response profile. In fact, there were no significant differences between conditions, but there was a significant bias across all conditions towards associating the vignette with suicide, despite the base rate being pre-determined at 50%. The bias was more pronounced in doctors and in male respondents. Moreover, many participants indicated substantial confidence in their decisions. The results are discussed in terms of availability bias and over-confidence bias.

## Introduction

Prevention of suicide is a health and social priority, and the importance of finding ways to reduce suicide rates has been re-iterated in many governmental directives, strategies and policy statements [1–3]. In the UK there are approximately 6000 suicides annually [4] accounting for approximately 1% of all-cause mortality [5]. Of these, approximately one quarter have been in contact with mental health services within the year preceding death [6] and so considerable obligation falls on Mental Health Professionals (MHPs) to identify patients most at risk.

There is a broad literature on the post-hoc social, clinical, occupational, demographic and biological associations of suicide e.g. [7–15] Although such studies make useful predictions about suicide incidence in the whole population, the associations and odds ratios provided by epidemiological studies and systematic reviews do not translate well to prescriptive models for the assessment of an individual case, primarily because suicide is, thankfully, a rare event.

Identifying and responding to risks is a driving factor in our mental healthcare system and the emphasis given to risk-based approaches has been evident in the literature for the last few decades [16–20]. An implicit assumption is that MHPs are proficient in understanding and recording risk information, yet some previous studies challenge this. For example, inter-rater reliability for risk assessment is often weak [21] and most MHPs have the same conceptual difficulties with probability that are found in the general population [22]. Categorical descriptors of patient risk, e.g., ‘high’, ‘medium’ or ‘low’, which are commonly used in psychiatric risk assessment tools, may convey quite different probabilities depending on the context in which they are used, or by whom they are read [23]. The way in which risk information is presented, for example, numerically or as percentages, may also bias perception and influence decision making [24]. And finally, two reviews of risk assessment proformas raise concerns about variability in the way such information is recorded and used across different UK mental health service providers [25, 26]. The process of completing risk assessments can be anxiety provoking for clinical staff, especially under the conditions of uncertainty that often prevail within psychiatric treatment settings. Uncertainty about future behaviours and events can rarely be eliminated, yet an adverse outcome for a patient who has already been risk assessed may have serious consequences for the treating clinician. For these reasons, some have argued that carrying out risk assessments can sometimes do more harm than good [27].

So given the inherent difficulties in assessing individual suicide risk, how do MHPs actually do this task? In the UK, NHS risk assessment training broadly follows an analytic approach, in which certain demographic and biographic features of a case (e.g. age, gender, marital status, diagnosis, previous incidents of self harm, admissions to hospital) are used collectively to help identify those at greater risk. This is, in essence, the approach that logically emerges from epidemiological data. In our experience though, clinicians often speak about more intuitive processes; they sense something about a patient that cannot be easily captured in a tick-box proforma.

We know that humans have a powerful, largely autonomous, ability to make inferences about emotion and intention based on physiological, sensory and behavioural cues (e.g. posture, movement, facial expression, prosody of speech). For example, facial expressions for the most common basic emotions (happiness, sadness, surprise, shock, anger and pain) are very robust across different cultures, suggesting a long-term evolution in communicating and recognising this information [28]. In the context of assessing risk we are interested to know whether automatic processing of such sensory information may sometimes compete with formal, more analytic, modes of reasoning. We hypothesized that information of a biographical nature might be interpreted differently in a risk-related decision task if there is competing or supportive sensory information available at the same time. In this study we chose to focus on the effect of facial information, as a first step towards examining a broader range of possible stimuli. Previous work has shown that the emotional state conveyed by briefly presented facial stimuli strongly affected favourability towards fictitious candidates in a simulated hiring task [29]. Similarly, when asked to evaluate the truth of advertising messages, participants were more likely to rate them as true when the message had been preceded by a subliminally presented face conveying a positive rather than negative emotion [30]. There is then at least some evidence that facial emotion information has the potential to induce decision bias.

This study was designed to test whether perception of a fictitious patient’s suicide risk is influenced by the presence of an associated face image and the specific emotion conveyed by the face. Furthermore, when the odds of suicide or non-suicide are presented as being equal, we explored whether a decision bias emerged in either direction. And finally we investigated the level of confidence ascribed by MHPs to their decision about the patient’s outcome. Previous research has shown that confidence in clinical judgements tends to increase with the level of information provided, even though the judgement itself may still be inaccurate [31].

## Method

### Design and Materials

This was an independent-groups study of MHPs comprising 4 conditions. Participants in each of the 4 conditions viewed the same concise vignette describing a fictitious young male with severe and enduring mental illness (see appendix for the full vignette). The vignette was devised by a consultant psychiatrist member of our research team, purely for the purpose of this study, and was piloted on a sample of MHP colleagues to ensure that it was not difficult to understand, nor unrepresentative of the kind of cases that MHPs would experience within their caseloads. It was not based on one specific real case but, rather, was written in such a way as to include clinical features that regularly occur within general adult psychiatry caseloads (e.g. bouts of depression, substance misuse, disorganised behaviour, hallucinations, difficulty holding down a job), but which were not in themselves highly predictive of suicidal ideation. Crucially, participants were instructed at the outset that this particular case report was drawn from a sample of twenty similar case reports collected as part of a recent audit on patient suicide, which included 10 people who had died by suicide and 10 people who had not. Participants were not told that the vignette was fictitious because we were concerned that respondents would pay less attention to the task if they believed that it was not real. Neither were they told whether the patient in the vignette did, or did not, commit suicide; indeed, their task was to make a judgement about which was most likely. By presenting the vignette in this context, we fixed the probability of suicide at 50%, this being the optimal point from which to measure bias in either direction.

Condition 1 served as the neutral, or control, condition: here, participants read the vignette without any associated picture and were asked to give their opinion about whether the patient in the vignette did or did not commit suicide. They were also asked to indicate the level of confidence ascribed to their decision by ticking one of 4 categories: (A) no confidence (50% chance of being correct, i.e. pure guess); (B) minimal confidence (65% chance of being correct); (C) substantial confidence (80% chance of being correct); (D) extreme confidence (95% or more chance of being correct, i.e. almost certain). Condition 2 was identical to condition 1 except that a small photograph depicting a young male with a moderately happy face was embedded within the vignette. We hypothesized that presenting a happy face alongside the vignette might induce a reduction in perceived risk below that associated with the control condition. In condition 3 the happy face was replaced by another photograph of the same individual male, but this time with an expression of moderate sadness. We predicted that a sad face would increase perception of risk above the level of the control condition. Finally, in condition 4, we used a third photograph depicting the same individual male, but this time with an expression of moderate anger. This condition was included because although anger may be seen as a negative emotion, previous research shows that angry looking faces are associated with greater competence and control than sad looking faces [32]. Hence we would predict a stronger association of suicide with the sad face (condition 3) relative to the angry face (condition 4). The photographs were drawn from the Binghampton University 3D Facial Expression database [33], and the first author obtained permission from the database team to use these. We did not use the most extreme levels of each expressed emotion available in this database because these tend to appear somewhat unnatural. Apart from the variation in photographs, the content and layout of both the vignette and the questions was identical across all 4 conditions.

Following from some of our previous work, which has found inter-professional variation in both attitudes towards risk assessment [34] and understanding of probability [22] we were also interested to see whether the professional background of our participants had any influence on their decisions and confidence levels. For example, doctors would usually have received a higher level of training in statistics and so they should, perhaps, be less inclined towards bias or over-confidence. And finally, we [21], and others [35] have previously reported gender differences in risk appraisal, so we examined whether any such differences emerged in the interpretation of the vignette.

We worked on the basis that if the facial expression were to exert a systematic influence on perception of suicide risk, then a sample size of 95+ per condition would provide 80% power in determining as significant, a difference of 40% and 60% between two conditions. We hypothesized that the ‘happy’ face condition might effect a decrease in the number of ‘suicide’ responses to 40%, while the ‘sad’ face might increase it to 60%.

### Participants

Participants were 400 MHPs working across three different counties (Hertfordshire, Bedfordshire and Essex). All were vocationally qualified staff working within a mental health setting (psychiatrists and staff-grade doctors, psychiatric nurses, or social workers). We limited participant inclusion to these 3 fairly broad groups because (i) we were confident of recruiting sufficient numbers within each, (ii) they have distinct training and education programmes prior to qualification, and (iii) all are required to assess patient risk routinely. In total, the study included: 104 psychiatrists and staff-grade doctors (51 female and 53 male, of mean age 41 ±10 years and of mean length of service 13 ±10 years); 240 psychiatric nurses (161 female and 79 male, of mean age 44 ±9 years and of mean length of service 16 ±11 years); 56 social workers (36 female and 20 male, of mean age 45 ±10 years and of mean length of service 11 ±9 years). These participants were split as evenly as possible across the 4 conditions. One hundred MHPs took part in each condition and were matched across condition on: (i) profession, (ii) gender distribution, (iii) age, and (iv) no. years NHS service (Table 1).

**Table 1:** Sample characteristics by condition. Doctors included consultant and staff grade psychiatrists.

Condition	1	2	3	4
Stimulus	*No Face*	*Happy Face*	*Angry Face*	*Sad Face*
Sample size (*n*)	100	100	100	100
Males: Females	39:61	38:62	37:63	38:62
Mean age (years ± SD)	42.1 (±10.2)	44.1 (±8.8)	43.2 (±10.2)	44.2 (±10.4)
Doctors (M:F)	26 (15:11)	26 (13:13)	26 (12:14)	26 (13:13)
Nurses (M:F)	60 (19:41)	60 (19:41)	60 (20:40)	60 (20:40)
Social Workers (M:F)	14 (5:9)	14 (6:8)	14 (5:9)	14 (4:10)
Mean Length of Service (years ± SD)	13.3 (±10.3)	14 (±10.6)	15.6 (±10.3)	14.9 (±11.2)

### Procedure

The National Research Ethics Service (NRES) for the UK National Health Service (NHS) gave ethical approval for this study. Opportunistic sampling was used initially and then, to ensure an even distribution of people from each professional background across the 4 conditions, we adopted a more purposive sampling method, thereby distributing questionnaires at events that were limited to specific professional groups. Although proper randomisation would be the favoured approach, it would have been very difficult to keep the study hypothesis secret from those participants tested within group settings and it would also have been difficult to co-vary the key demographic variables, given that we employed a simple categorical outcome measure. Questionnaires were generally distributed at team meetings, seminars, educational and training events, and all participants completed them on a voluntary basis and not in the presence of any of the authors. Respondents were asked to generate an anonymity code based upon letters derived from personal details, allowing us to exclude duplicate returns from the same individuals (these were minimal in number). When handed out at meetings or training events, we were careful not to mix questionnaires from different conditions. Incomplete questionnaires were excluded from analysis.

## Results

### 1. Interpreting The Vignette: The effect of Facial Stimuli

Across all 400 participants, there was an overall bias towards associating the vignette with suicide (230 ‘suicide’ vs. 170 ‘non-suicide’ judgements; 58% vs. 42%, Chi-square *df*1 = 9, *p* < 0.005). However, although there was some variation in how respondents categorised the vignette across conditions (Table 2), this did not reach statistical significance (Chi-square, *df*3 = 2.33, *p* > 0.5). It is interesting to note, however, that the condition generating the greatest number of ‘suicide’ responses was actually the one that included a happy face. This clearly runs contrary to our earlier predictions.

**Table 2:** ‘Suicide’ and ‘Non-suicide’ responses by condition.

Response	*No Face*	*Happy Face*	*Angry Face*	*Sad Face*	*All*
Suicide	59	63	54	54	230
No Suicide	41	37	46	46	170
Both	100	100	100	100	400

We also carried out separate analyses to compare across conditions for each of the 3 professional groups but there were no significant differences: Doctors (Chi-square *df*3 = 1.02, *p* > 0.7); Nurses (Chi-square *df*3 = 3.64, *p* > 0.3); Social Workers (Chi-square *df*3 = 0.29, *p* > 0.9). Neither were there any differences across the 4 conditions when male respondents (Chi-square *df*3 = 1.17, *p* > 0.7) and female respondents (Chi-square *df*3 = 3.86, *p* > 0.25) were analysed separately. In short, the presence or absence of a facial stimulus, and the emotional expressed therein, did not influence how participants responded.

### 2. Judgement About The Vignette: The effect of Profession and Gender

Interestingly the bias towards associating the vignette with an outcome of suicide was most evident for the Doctor group, with respective rates for Doctors, Nurses and Social Workers across all 4 conditions combined being: 68/104 (65.4%), 132/240 (55%) and 30/56 (53.6%). This difference was highly significant (Chi-square *df*2 = 18.1, p < 0.0001).

We also examined whether the gender of the respondent had any bearing on suicide judgement. Across all conditions, 102/152 (67.1%) male respondents associated the vignette with suicide, compared with 128/248 (51.6%) females, a difference that was also highly significant (Chi-square *df*1 = 8.63, *p* < 0.005).

Since there were considerably more females in both the Nurse and Social Worker groups, but not in the Doctor group (see Table 1), and given that a gender bias emerged, it is possible that the reported difference between professional groups may be attributable, partly or wholly, to the gender distribution of participants. For this reason we cross-tabulated gender differences with profession for those participants who selected ‘suicide’ (Table 3).

**Table 3:** ‘Suicide’ responses for male and female participants by professional group.

Professional Group	Male	Female
Doctors	36 (68%)	32 (63%)
Nurses	54 (68%)	78 (48%)
Social Workers	12 (60%)	18 (50%)

The percentage scores in Table 3 suggest that the gender and professional biases may occur somewhat independently of each other. However this could not be tested statistically because it only includes those respondents who selected the ‘suicide’ response. To analyse the association and interaction between professional group, gender and response type is not possible for categorical data. A male bias is numerically evident in all 3 professional groups but is smallest in the doctor group, yet there is a noticeable difference between the female doctors relative to females in the other 2 professional groups. The age of the respondent was not associated with the response given (43.3 ±9.9 vs. 43.4 ±10, *t* = 0.175, *p* = 0.86), and neither was length of service (14.8 ± 10.9 vs. 13.9 ±10.2, *t* = 0.879, *p* = 0.38).

So far we have examined several variables that we thought might be associated with how MHPs interpret the vignette. However we also asked our participants to indicate the level of confidence they would ascribe to their decisions. This is important because if all participants were to indicate that they had no confidence at all in their response (i.e. they always selected category A), then it would be much less meaningful to talk about inter-professional and gender differences in decision-making.

### 3. Confidence Ratings

Confidence ratings were represented by 4 categories, each one comprising a semantic label and a probability (%). To recap, these were: (A) ‘Not confident at all—approx. 50% chance of being right’; (B) ‘Minimally confident–approx. 65% chance of being right’; (C) ‘Substantially confident—approx. 80% chance of being right’; and (D) ‘Extremely confident—approx. 95% chance or more, of being right’. Table 4 shows the distribution of confidence scores attributed to the two possible outcomes.

**Table 4:** Confidence ratings by condition and by response.

Confidence	A: 50%	B: 65%	C: 80%	D: 95+%	*n*
*Suicide*	32 (14%)	93 (40%)	100 (44%)	5 (2%)	230
*No Suicide*	24 (14%)	68 (40%)	74 (44%)	4 (2%)	170
**Grand Total**	**56 (14%)**	**161 (40%)**	**174 (44%)**	**9 (2%)**	**400**

The modal response is C, closely followed by B, while categories D and A were much less frequently endorsed. There was minimal variation across the 4 different face conditions and so we have collapsed the confidence data across all conditions in Table 4. Degree of confidence is not affected by the presence or type of face image, nor the decision response (suicide or non-suicide). For this reason, the remaining analyses on confidence ratings were collapsed across facial condition and response. Table 5 shows the distribution of confidence ratings according to professional group and gender.

**Table 5:** The Distribution of Confidence Ratings by Profession and Gender.

Confidence	A: 50%	B: 65%	C: 80%	D: 95+%	*n*
*Doctor*	24 (23%)	41 (39%)	37 (36%)	2 (2%)	104
*Nurse*	23 (10%)	87 (36%)	124 (52%)	6 (2%)	240
*Social Worker*	9 (16%)	33 (61%)	13 (24%)	1 (2%)	56
*Males*	22 (14%)	63 (41.5%)	63 (41.5%)	4 (3%)	152
*Females*	34 (14%)	98 (39%)	111 (44%)	5 (2%)	248

Although we found earlier that the gender of the respondent was associated with the response choice made, the confidence associated with that decision appears not to be. There is some variation according to profession, with doctors tending to endorse the no confidence category (A) more than the other groups, and nurses tending towards greater confidence, but on the whole *all* 3 professional groups were far more likely to choose categories B or C. Degree of confidence was not significantly associated with length of service (*F df* 3, 399 < 1).

## Discussion

We have investigated, using a fictitious case study, some factors that may influence suicide risk perception in psychiatrists, nurses and social workers. The vignette we have used is not comparable with the kind of interaction that MHPs have with real patients. However, MHPs are required to assess patient risk under some level of uncertainty, and so we have tried to capture some elements of this here, albeit within a somewhat contrived scenario.

Although long-term mental illness is a known risk factor in completed suicide, the base rate of suicide is low and so this tragic outcome is still a relatively rare event for most MHPs. Reviews and epidemiological studies highlight several factors that increase the risk of suicide e.g. [14] but these factors are actually much more predictive of non-suicide. Thus suicidal behaviour is notoriously difficult to predict on an individual basis. Nonetheless, because of its gravity, this outcome has received huge attention in the risk prevention policy and literature.

In our fictitious vignette, we were careful to include biographical information fairly typical of a case seen within general adult psychiatry, but not highly associated with death by suicide (i.e., we avoided mentioning previous suicide attempts, self harm or suicidal ideation). Moreover we presented the vignette in the context of an audit examining an equal number of suicide and non-suicide cases, thereby fixing the likelihood of either outcome at 50%. Our interest was in determining which of several factors might bias respondents towards, or away, from believing the case was associated with suicide. By including images of different facial expressions, we explored whether information of a more sensory nature might alter the perception of risk. It is well known that human perceptual and attention systems are biased towards facial stimuli, and that facial emotion can introduce bias into certain decision tasks [29, 30], so we hypothesized that facial expressions might influence the interpretation of information within the vignette. In actual fact this was not the case, or at least it was not demonstrable within the limitations of this experiment. There was some variation across conditions but not to a significant degree and, if anything, the observed pattern ran in the opposite direction to what we had initially predicted. It is possible that the simple categorical outcome measure used here (suicide vs. non-suicide) is simply not powerful enough to detect any effect and also that the stimulus (a static picture of a face) was insufficient to model a real-life situation where the dynamics of facial movement, posture, gesture and prosody of speech are all available to the observing clinician. It is also possible that the image itself, being drawn from a databank, may not seem clinically plausible to experienced professional observers.

In distinction to the null finding for facial stimuli, a clear bias emerged towards associating the vignette with an outcome of suicide. Given that the vignette was framed to give equal probability (50%) to either outcome, the normative response across the whole group of participants should be a roughly equal assignation to each category, while for any given individual, the expected response should be either of the outcome categories but with zero confidence expressed about the decision. The results do not tell us what the mechanisms of bias might be. Representativeness bias seems an unlikely explanation because although the features of this vignette may well be associated with some cases of suicide, it is arguable that they are much more strongly associated with non-suicide and especially so for people who regularly work in mental health treatment settings. Availability bias involves assignation of an object or event to a category because instances of that category are more easily brought to mind. This may be the case here because when suicides occur, they are usually dramatic, emotive, and often highly publicised events, whereas the ‘non-suicide’ category may not have the same pull. It is intriguing that doctors were significantly more prone to this bias. If the overall bias towards the suicide outcome was related to using availability heuristics, then it is possible that doctors are more prone to this because they have greater role and responsibility in the documentation and subsequent enquiries in cases of completed suicide and this decision task may have brought to mind experiences of attending a coroner’s court. This may also give greater emotional valence to suicide for this particular professional group, although we have no evidence yet in support of this. It is similarly intriguing that such a strong male bias was found. Previous studies of risk taking and risk assessment have demonstrated strong gender differences, with the prevailing view being that females tend to be more cautious than males in appraising risk e.g. [35]. In associating the vignette with an outcome of suicide, male participants are arguably exhibiting a greater level of caution/concern than females. We did not design this study to specifically examine gender effects but given that such a strong bias emerged, it would be interesting to see if this pattern is replicated and it would also be interesting to introduce a female version of the vignette in future work too.

Perhaps the most noteworthy finding in the study is the overconfidence exhibited by MHPs in their decisions. Given the way in which the information was framed, Category A (not confident at all) should have been selected by the vast majority of respondents. However it was endorsed by just 14%, suggesting that the remaining 86% were influenced by information they read in the vignette and used this as the basis for their decision. That well over 40% participants indicated that they were substantially or extremely confident in their decision is quite worrying and re-iterates a finding from our previous work that MHPs have conceptual difficulties in dealing with probabilistic information [22]. Overconfidence bias, which can be broadly defined as being more certain about a matter than the facts allow, is a widespread human psychological feature, well reported over many years. What this experiment suggests is that MHPs are just as easily drawn into an over confident view of their opinions as the human mind is generally. This is not without clinical significance, for if we are to rely on MHPs to make normatively and analytically correct estimations of risk then that should also include some calibration for the degree of certainty with which a particular opinion can be held. We deliberately contextualised the vignette in a way that placed the risk of suicide and non-suicide at equipoise. Clearly such odds would be highly unusual within psychiatric cases and so the known pre-test likelihood of 50% may have skewed decision making, especially in relation to an outcome where the costs of a false negative are so much greater than a false positive. Furthermore, the decision task in this study involved a retrospective review of a case report where the outcome was ostensibly known, albeit hidden from the study participants; the decision made by the respondent would have no bearing on real outcomes and so the lack of accountability for the decision may have inflated confidence levels. Nonetheless, it is still difficult to understand why so few MHPs endorsed the no confidence option.

Given the great weight ascribed to risk assessment in the treatment and care of people with mental illness, there are good reasons to pursue further research in this area. The reliability, validity and utility of risk assessment cannot be unquestionably accepted and, as noted previously, there may be a negative impact on those MHPs who have to undertake them [27]. Furthermore, the low sensitivity and specificity of risk assessment tools, especially in the context of self-harm, has been noted previously [36, 37] and it is clear that proper assessment of suicide risk requires a thorough psychosocial assessment. At a more general level, without a greater understanding of the psychology and limitations of risk estimation by clinicians, it is difficult to see how meaningful teaching can be given to the professionals who are expected to do this task on a daily basis.

## Appendix: The Vignette

You are invited to take part in a study investigating the perceptions of Mental Health Professionals in the context of patient suicide. There is no obligation to take part but we would be very grateful if you do feel able to do so. If you are willing to take part, *please read the following information carefully* and answer the questions at the end. The task should take no more than 5 minutes and your responses will remain completely anonymous. The data collected from this study will not be used to make inferences about specific individuals: we are interested only in general patterns of opinion.

Presented below is a case report of a patient. This is one of 20 reports that were collected as part of a recent audit on patient suicide in the UK. This audit focused on 20 patients with severe and enduring mental illness, of which half committed suicide and half did not.

In this exercise we ask you to give your opinion about whether the case-report below relates to one of the patients who killed themselves or to one of the patients who did not kill themselves. We are interested in your honest opinion and there are no trick questions. After you have read the case-report please answer the questions overleaf.

### Case Report (1 of 20)

The patient, a Caucasian male, was born in 1985. His parents separated when he was a baby and his mother brought him up alone. His mother worked as a catering supervisor. She was of a caring nature, if somewhat over-protective of her son. He completed mainstream education, but academic achievement was poor because of increasing substance abuse during his teens.

He first received psychiatric attention for depression as an outpatient at the age of 18. One year later he was admitted to a psychiatric ward for delusions, hallucinations and disorganized behaviour. Progress under treatment was broadly favourable: symptoms fully resolved and after a period of time he became apprenticed as a motor mechanic. Although denying that he had any mental illness, he was co-operative with whatever treatment his psychiatrist advised.

Although initially remaining free of mental illness, escalating substance abuse, mainly cannabis and alcohol, led him to lose his apprenticeship and to be ejected from the home by his mother. However, he managed to set himself up in a flat on his own, stay in contact with a limited circle of friends and to support himself doing odd jobs for cash-in-hand. A good relationship with his social worker developed; advice from her being available when needed, from time to time. After the hospitalisation at the age of 18 few girls seemed interested in him and he came to feel inadequate in comparison to other young men.

Three years after the first hospitalisation there was insidious decline into a persistently deluded, hallucinated and disorganised state. Three months of further treatment in hospital produced only partial benefits—about a 50% reduction in symptom intensity. He was discharged to supported accommodation with daily visits from members of the community psychiatric team. Substance abuse recurred quickly after discharge, prompting the treating psychiatrist to advise the patient of a poor prognosis if abstinence could not be achieved. During the three years between first and second hospitalisations his mother had developed arthritis that significantly limited her ability to assist in her son’s care.

___________________________________

Now we would be very grateful if you would answer the questions:

**Question 1:** In your opinion, does this case-report most likely relate to (please tick either A or B as appropriate):

1. One of the patients that did commit suicide; or
2. One of the patients that did not commit suicide

**Question 2:** How confident do you feel about your opinion? (please tick one response below)

1. not confident at all: e.g. approximately 50% chance of being right
2. minimally confident: e.g. approximately 65% chance of being right
3. substantially confident: e.g. approximately 80% chance of being right
4. extremely confident e.g. approximately 95% chance, or more, of being right

## References

[1] Department of Health (1999) National Service Framework for Mental health: Modern standards and service models. London: DH.

[2] Department of Health (2002) National Suicide Prevention Strategy for England. London: DH.

[3] Department of Health (2012) Preventing Suicide in England: A cross-government outcomes strategy to save lives. London: DH.

[4] Office for National Statistics (2014) Suicides in the United Kingdom, 2012 Registrations, ONS. Available: http://www.ons.gov.uk/ons/rel/subnational-health4/suicides-in-the-united-kingdom/2012/index.html

[5] Brock A, Baker A, Griffiths C, Jackson G, Fegan G, Marshall D (2006) Suicide trends and geographical variations in the United Kingdom, 1991–2004. Health Statistics Quarterly, 31, 6–22. 16972691

[6] Appleby L, Shaw J, Sherratt J, Amos T, Robinson J, McDonnell R, et al (2001) Safety First, Summary Report of the National Confidential Inquiry into Suicide and Homicide by People with Mental Illness. London: Stationery Office.

[7] Angst J, Angst F, Stassen HH (1999) Suicide risk in patients with major depressive disorder. J Clinical Psychiatry, 60, 57–62. 10073389

[8] Cassells C, Paterson B, Dowding D, Morrison R (2005) Long- and short-term risk factors in prediction of inpatient suicide: a review of the literature. Crisis, 26(2), 53–63. 16138741 10.1027/0227-5910.26.2.53

[9] Mortensen PB, Agerbo E, Erikson T, Qin P, Westergaard-Nielsen N (2000) Psychiatric illness and risk factors for suicide in Denmark. Lancet, 355, 9–12. 10615884 10.1016/s0140-6736(99)06376-x

[10] Nordstrom P, Samuelsson M, Asberg M, Traskman-Bendz L, Aberg-Wistedt A, Nordin C, Bertilsson L (1994) CSF5-HIAA predicts suicide risk after attempted suicide. Suicide And Life Threatening Behaviour, 24, 1–9. 7515519

[11] Hall RCW, Platt DE, Hall RCW (1999) Suicide risk assessment: a review of risk factors for suicide in 100 patients who made severe suicide attempts: evaluation of suicide risk in a time of managed care. Psychosomatics, 40, 18–27. 9989117 10.1016/S0033-3182(99)71267-3

[12] Qin P, Agerbo E, Bo Mortensen P (2003) Suicide risk in relation to socioeconomic, demographic, psychiatric, and familial factors: A national register–based study of all suicides in Denmark, 1981–1997. American Journal of Psychiatry, 160, 765–772. 12668367 10.1176/appi.ajp.160.4.765

[13] Lorant V, Kunst AE, Huisman M, Costa G, Mackenbach J. (2005) Socio-economic inequalities in suicide: a European comparative study. British Journal of Psychiatry, 187, 49–54. 15994571 10.1192/bjp.187.1.49

[14] Hawton K, Casanas I, Cornabella C, Haw C, Saunders K (2013) Risk factors for suicide in individuals with depression: a systematic review. Journal of Affective Disorders, 147(1–3), 17–28. 10.1016/j.jad.2013.01.004 23411024

[15] Roberts SE, Jaremin B, Lloyd K (2013) High-risk occupations for suicide. Psychological Medicine, 43(6), 1231–1240. 10.1017/S0033291712002024 23098158 PMC3642721

[16] Baldwin D, Mayers A, Elgie R (2003) Suicide in psychiatric inpatients. Clinical Risk, 9(6), 229–232.

[17] Bouch J, Marshall JJ (2005) Suicide risk: structured professional judgement. Advances in Psychiatric Treatment, 11, 84–91.

[18] Brunton K (2005) The Evidence on how nurses approach risk assessment. Nursing Times, 101(28), 38–39. 16048000

[19] Morgan S (2000) Clinical Risk Management: A Clinical Tool and Practitioner Guide. London The Sainsbury Centre for Mental Health.

[20] Monkley-Poole S (1998) Calculating risk in community mental health nursing; the decision analysis approach. Mental Health Care, 21(2), 56–59.

[21] Gale TM, Woodward A, Hawley CJ, Hayes J, Sivakumaran T, Hansen G (2002) Risk assessment for people with mental health problems: a pilot study of reliability in working practice. International Journal of Psychiatry in Clinical Practice, 6, 73–81. 10.1080/136515002753724063 24931932

[22] Gale TM, Hawley CJ, Sivakumaran T (2003) Do mental health professionals really understand probability? Implications for risk assessment and evidence-based practice. Journal of Mental Health, 12(4), 417–430.

[23] Gale TM, Hawley CJ, Sivakumaran T (2011) Classification of risk in psychiatry. Psychiatria Danubina, 23, 198–202. 21894135

[24] Jefferies-Sewell K, Sharma S, Gale T, Hawley CJ, Georgiou G, Laws KR (2015) To admit or not to admit? The effect of framing on risk assessment decision making in psychiatrists. Journal of Mental Health, 24(1), 20–23. 10.3109/09638237.2014.951477 25188819

[25] Hawley CJ, Littlechild B, Sivakumaran T, Sender H, Gale TM, Wilson KJ (2006) Structure and content of risk assessment proformas in mental healthcare. Journal of Mental Health, 15(4), 437–448.

[26] Higgins N, Watts D, Bindman J, Slade M, Thornicroft G (2005) Assessing violence risk in general adult psychiatry. Psychiatric Bulletin, 29, 131–133.

[27] Undrill G (2007) The risks of risk assessment. Advances in Psychiatric Treatment, 13, 291–297.

[28] Ekman P, Friesen WV (1971) Constants across cultures in the face and emotion. Journal of Personality and Social Psychology, 17 (2), 124–129. 5542557 10.1037/h0030377

[29] Skandrani-Marzouki I, Marzouki Y (2010) Subliminal emotional priming and decision making in a simulated hiring situation. Swiss Journal of Psychology, 69 (4), 213–219.

[30] Channouf A (2000) Subliminal exposure to facial expressions of emotion and evaluative judgments of advertising messages. European Review of Applied Psychology, 50 (1), 19–25.

[31] Oskamp S (1965) Over-confidence in case-study judgements. Journal of Counselling Psychology, 29(3), 261–265. 10.1037/h0022125 14303514

[32] Tiedens LZ (2001) Anger and advancement versus sadness and subjugation: the effect of negative emotion expressions on social status conferral. Journal of Personality and Social Psychology, 80(1), 86–94. 11195894

[33] Yin L, Wei X, Sun Y, Wang J, Rosato MJ (2006) A 3D facial database for facial behaviour research. 7th International Conference on Automatic Face and Gesture Recognition, 10–12 April, 211–216.

[34] Hawley CJ, Gale TM, Sivakumaran T, Littlechild B (2010) Risk assessment in mental health: staff attitudes and an estimate of time cost. Journal of Mental Health, 19(1), 88–98. 10.3109/09638230802523005 20380501

[35] Harris CR, Jenkins M, Glaser D (2006) Gender differences in risk assessment: why do women take fewer risks than men? Judgement and Decision Making, 1(1), 48–63.

[36] NICE (2011) Self-harm in over 8s: long term management https://www.nice.org.uk/guidance/cg133

[37] Quinlivan L, Cooper J, Steeg S, Davies L, Hawton K, Gunnell D, Kapur N (2014) Scales for predicting risk following self-harm: an observational study in 32 hospitals in England. BMJ Open. http://bmjopen.bmj.com/content/4/5/e004732.full 10.1136/bmjopen-2013-004732 PMC4025469 24793255

